# Natural history of *Javeta pallida* Baly, 1858 on *Phoenix* palms in India (Chrysomelidae, Cassidinae, Coelaenomenoderini)

**DOI:** 10.3897/zookeys.597.6876

**Published:** 2016-06-09

**Authors:** Koormath Mohammed Shameem, Kaniyarikkal Divakaran Prathapan, Mannankadiyan Nasser, Caroline Simmrita Chaboo

**Affiliations:** 1 Department of Zoology, University of Calicut, Malappuram 673 635, Kerala, India; 2 Department of Entomology, Kerala Agricultural University,Vellayani P.O., Trivandrum 695 522, Kerala, India; 3 Department of Zoology, University of Calicut, Malappuram 673 635, Kerala, India; 4 Division of Entomology, Natural History Museum, and Department of Ecology and Evolutionary Biology, 1501 Crestline Dr., Suite 140, University of Kansas, Lawrence, KS, 66049–2811, USA

**Keywords:** Leaf beetles, leaf miner, pest, Arecaceae, Eulophidae, oil palm

## Abstract

Members of the Old World hispine tribe, Coelaenomenoderini, are documented on host plants of Arecaceae, Cyperaceae, and Zingiberales. A few species are renowned pests of oil palm, especially in Africa. The host plants and natural history of *Javeta pallida* Baly, 1858, the only Indian species of the tribe, is reported for the first time. These beetles can densely infest indigenous wild date palms, *Phoenix sylvestris* (L.) Roxb. (Arecaceae), and also use the introduced date palm, *Phoenix dactylifera* L., which is an expanding crop in India. *Javeta* females lay single eggs and cover each with an ootheca. All larval stages mine the leaves and pupation occurs within the larval mine. Adults are exophagous, leaving linear feeding trenches. Natural and induced infestations of *Javeta pallida* on these two palms were observed and the potential of *Javeta pallida* as a pest of date palm in India is discussed. *Javeta pallida* completed development on *Phoenix* palms in 52–88 days (mean 66.38 days) with egg period 11–15 days (mean 12.8 days), larval period 21–54 days (mean 33.02 days) and pupal period 17–23 days (mean 20.52 days). *Elasmus longiventris* Verma and Hayat and *Pediobius imbreus* Walker (Hymenoptera: Eulophidae) parasitize the larva and pupa of *Javeta pallida*.

## Introduction

**Figures 1, 2.**
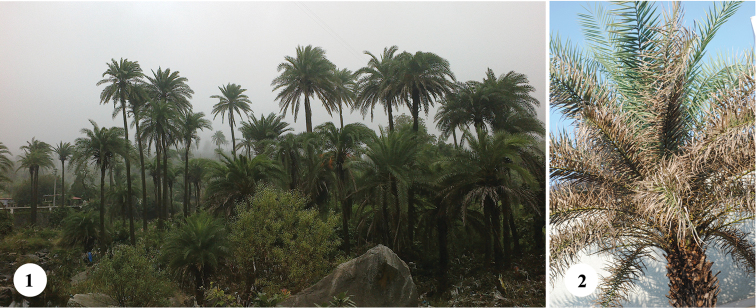
*Phoenix* palms in India. **1** Naturally growing *Phoenix sylvestris* in Mount Abu, Rajasthan **2** *Javeta pallida* infested *Phoenix sylvestris*, Tirurangadi, Kerala.

The palm genus *Phoenix* L. (Arecaceae: Phoeniceae) comprises 15 species which are grown as ornamentals and for food and beverage. The sweet fruit of several species are eaten and sap is tapped to make various fermented drinks and vinegar. Nine *Phoenix* species occur in southern Asia (Henderson 2009; Govaerts et al. 2015). *Phoenix sylvestris* (L.) Roxb., the silver date palm, the wild date palm or the date sugar palm, is a medium-sized palm with solitary stems up to 20 m in height (Fig. 1) (Henderson 2009). According to Krishnamurthi et al. (1969), about 29 million palms of *Phoenix sylvestris* exist in India; they summarized its biology, cultivation practices and myriad local uses in the encyclopedic Wealth of India Series. Banerji (2012) discussed the wild date palm and the near-mythical status of the palm sugar in Bengali gastronomy (West Bengal state in India and the adjoining area of Bangladesh that form the erstwhile Bengal). Thirteen species of insect herbivores have been documented on *Phoenix sylvestris* (Mathur and Singh 1961; Howard et al. 2001).

The date palm, *Phoenix dactylifera* L. is one of the first cultivated tree crops, being grown since early Bronze Age (late 4th/early 3rd millenia B.C.) (Tengberg 2012). Date palm is commercially grown in Gujarat and Rajasthan in India (Radha and Mathew 2007). Despite the popularity of its fruit, date palm is not cultivated in Kerala, India (where the outbreak of the insect was noticed), due to unfavorable climatic conditions. Stray seedlings, which germinate from the seeds discarded after eating the flesh, are rarely observed in Kerala. Carpenter and Elmer (1978) reviewed pests and diseases of *Phoenix dactylifera* globally. In India, about 21 insect pests are associated with the species (Mathur et al. 1958; Mathur and Singh 1961; Wadhi and Batra 1964; Batra 1972; Bindra and Varma 1972; Sohi and Batra 1972; Batra and Sohi 1974; Sachan 1976; Muralidharan 1993; Radha and Mathew 2007).

The Old World “hispine” tribe Coelaenomenoderini comprises nine genera and 88 species (Gressitt and Kimoto 1963; Gressitt and Samuelson 1990; Würmli 1975; Staines 2012b). The limited data indicates Arecaceae, Cyperaceae, Pandanales and Zingiberales as host plants (Staines 2004, 2012b). Juvenile stages (larva or pupa) are known for just two species—Coelaenomenodera (Coelaenomenodera) elaeidis Maulik (Maulik 1920; Cox 1988, 1994) and *Cyperispa hyloytri* Gressitt (Cox 1996).

Some species are pests of oil palm, *Elaeis guineensis* Jacq. (Rajagopalan and Alderungboye 1970; Calvez 1976; Godfray and Chan 1990; Mariau and associates 1972–2004; Cochard et al. 2005). *Coelaenomenodera* Maulik is by far the best-known genus because three species are significant pests of oil palm in Africa and have received much research attention, especially by the French agro-entomologist, Dominique Mariau. Mariau and colleagues intensely studied Coelaenomenodera (Coelaenomenodera) elaeidis Maulik for over 10 years as it was considered the most important pest of oil palm in West Africa (Morin and Mariau 1970). Due to the mining behavior, palm leaflets are severely damaged and produce lower yields (Ruer 1964) by as much as a 30% reduction (Simmonds 1970). The biology, life cycle and enemy complex are well-documented for Coelaenomenodera (Coelaenomenodera) elaeidis (see Maulik 1920; Cotterell 1925; Waterston 1925; Cachan 1957; Morin and Mariau 1970, 1971, 1974; Mariau and Morin 1971, 1972, 1974; Mariau 1976, 1999; Mariau et al. 1978; Bernon and Graves 1979; Philippe et al. 1979; Mariau and Philippe 1983; Philippe 1990; Timti 1991; Mariau et al. 1999a), Coelaenomenodera (Coelaenomenodera) lameensis Berti and Mariau (see Berti and Mariau 1999; Mariau and Lecoustre 2000, 2004; Mariau 2001), Coelaenomenodera (Coelaenomenodera) perrieri Fairmaire (Mariau 1988, 2001; Lecoustre et al. 1980), and Coelaenomenodera (Coelaenomenodera) speciosa Gestro (Uhmann 1961; Santiago-Blay 2004). These provide a model for research on other coelaenomenoderine species which might pose pests of economically-valuable palms.

*Javeta pallida* Baly, 1858, the type species of the genus, is the only species of Coelaenomenoderini known from India (Maulik 1919). *Javeta* Baly, 1858 comprises 19 species found in Asia (Staines 2012a, b). The biology of *Javeta* is poorly known but records indicate host associations of three species with Arecaceae (Jolivet 1989; Jolivet and Hawkeswood 1995; Santiago-Blay 2004)—*Javeta arecae* Uhmann, 1943 on *Areca catechu* (Uhmann, 1943) and *Areca* sp. (pinang; Kalshoven 1981); *Javeta corporaali* Weise, 1924 on *Pinanga kuhlii* Blume (Uhmann 1955); and *Javeta thoracica* Uhmann, 1955 on *Areca* sp. (Uhmann 1955) and *Metroxylon* sp. (Kalshoven 1957). Steiner (2001) listed undetermined species of *Javeta* amongst the insects associated with the rattan palms, *Daemonorops hirsuta* Blume and *Calamus manan* Miq. (Arecaceae: Calameae). Data on *Javeta* juvenile stages is limited to the described pupa of *Javeta corporaali* by Uhmann (1955) and the mining larva of *Javeta arecae* (Kalshoven, 1981). The only information on *Javeta* life history is a short remark by Kalshoven (1951: 759; 1981: 456) about *Javeta arecae*,﻿ reported from an outbreak in Sumatra: “The larvae make long mines in the leaves and feeding by the beetles produces brown stripes”. No information is available on the egg, oviposition and pupation sites.

The goal of this paper is to report the host plants and natural history of *Javeta pallida* for the first time, taking advantage of a heavy infestation on *Phoenix sylvestris* in southern India (Fig. 2). *Javeta pallida* was originally described from the Nilgiri Hills, southern India, and is known today to extend to West Bengal and Uttar Pradesh in north India (Basu 1999). Our discovery of the heavy infestation has implications for the cultivation of two regional palm food resources, both the indigenous local host and the date palm, *Phoenix dactylifera* introduced to India. Thus, the propensity of coelaenomenoderine species to become significant pests of major palm crops in tropical countries and the lack of information on the biology of *Javeta pallida* motivates this research contribution. We use natural populations and transfer experiments to: 1) study the life cycle and assemble a specimen collection for morphological study, 2) explore the potential of *Javeta pallida* to become a pest of the date palm in India, and 3) compare beetle development on the two hosts.

## Material and methods

The study is based on field observations of live populations of *Javeta pallida* at Malappuram District, north Kerala, India, led by authors KMS, PKD, and MN. To document the life cycle and biology, beetles were reared on date palm, *Phoenix dactylifera* and on the wild date palm, *Phoenix sylvestris*.

### Field sites

(i) The initial infestation of *Javeta pallida* was observed on three stray palms of *Phoenix sylvestris* during December, 2014 (Fig. 2). The plants are ornamentals in a 30 m wide “garden” between a concrete building and a road joining the National Highway 17 at Tirurangadi (N11°02'12.0", E75°56'12.6", 47m above msl).

(ii) Remnants of natural infestation was observed on a stray date palm of about ten years old at Tirurangadi (N11°02'17.24", E75°55'40.61", 35m above msl) in April, 2015.

(iii) Rearing of *Javeta pallida* on *Phoenix dactylifera* was carried out at Tirurangadi (N11°02'31. 60", E75°55'8.72", 23m above msl) on a three-year old stray date palm.

(iv) Rearing of *Javeta pallida* on *Phoenix sylvestris* was carried out at the Botanical Garden of the Calicut University, Kerala (N11°07'59.01", E75°53'22.83", 77m above msl) on a 10–12 year old, 2.25m tall palm (excluding crown).

### Rearing of *Javeta pallida* on *Phoenix dactylifera*

**Dry season.** Nine adults were released and confined with pieces of nylon net (mesh size 0.701mm–0.827 x 0.628–0.686mm; Nylon Maharani Net http://www.indiamart.com/goldfinchcreators/fabrics.html) on a frond on 23.IV 2015 for two days. On a second frond of the same palm, five adults were confined on 27.IV 2015 and a sixth adult was added on 30.IV.2015. The beetles were maintained on the frond until 3.V.2015.

**Rainy season.** During the rainy season, seven adults were confined on a third frond of the same palm of *Phoenix dactylifera* on 10.VI.2015 and five more were added on the next day. All of them were retained on the frond till the eighth day. On a fourth frond, seven adults were confined on 17.VI.2015, and were retained till 20.VI.2015.

### Rearing of *Javeta pallida* on *Phoenix sylvestris*

Rearing was carried out only during rainy season on *Phoenix sylvestris*. Three young fronds were selected and 12 adults were used in the study. On the first frond, 12 adults were confined with nylon net for five days from 10.VI.2015. The same adults were shifted to the second frond on 15.VI.2015 and confined for two days. They were again shifted to a third frond on 17.VI.2015 and confined on it for a single day.

All adults used in rearing experiments were collected from the wild population of *Javeta pallida* on *Phoenix sylvestris* at the first field site in Tirurangadi.

Individual eggs were counted and marked on the leaflets every day and the development was followed through larva and pupa till the emergence of adult. Developmental periods such as egg, larval and pupal duration of all individuals, which could be tracked, were recorded. The date of hatching of the eggs was determined by observing the beginning of the leaf mine (Fig. 5). Similarly the end of the larval period was determined by observing cessation of feeding followed by the withdrawal of the mature larva from the leading end of the mine. After pupation, the leaflets holding the pupa in larval mine, were removed from the leaf rachis and were placed individually inside the bottles for emergence of adults.

**Figures 3–14.**
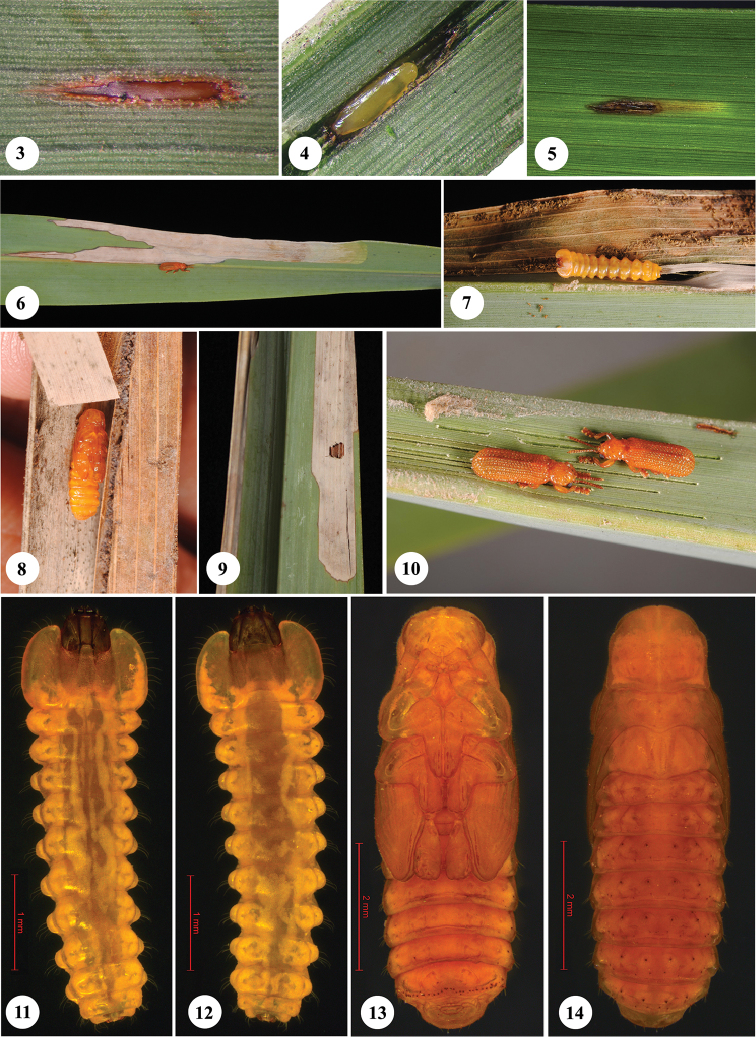
Life stages of *Javeta pallida*. **3** Egg covered with ootheca **4** Egg, ootheca removed **5** Beginning of leaf-mine **6** Leaf mine and adult of *Javeta pallida* **7** Larva in leaf-mine, exposed **8** Pupa in leaf-mine, exposed **9** Adult exit hole **10** Adults and feeding trenches **11** Larva, ventral view **12** Larva, dorsal view **13** Pupa, ventral view **14** Pupa, dorsal view. (Figs 3–10 on *Phoenix sylvestris*, except 9 on *Phoenix dactylifera*).

Mean values of developmental periods of individuals reared during dry season (eggs laid in April, 2015) and rainy season (eggs laid in June, 2015) on *Phoenix dactylifera* were compared using t-test of significance (Panse and Sukhateme 1985). Similarly, the developmental periods of individuals reared during rainy season on *Phoenix dactylifera* and *Phoenix sylvestris* were compared using the same tool to find out possible statistical difference in developmental periods on the two host species of *Phoenix*.

**Visits to commercial plantations.** Two visits, during January and April, 2015, were made to the commercial plantations of date palms in Dharmapuri, Tamil Nadu, southern India.

**Collection of natural enemies.** Naturally infested leaves of *Phoenix sylvestris* from the first field site were brought to the laboratory and kept in plastic containers of about 5 L capacity for emergence of adult parasitoids.

**Specimen collection.** A total of 173 adults, 81 pupae, 41 larvae, and nine eggs were collected at Tirurangadi on 12, 14, and 28.XII.2014 (KMS and KDP); one adult was collected at Jakkur Lake, Bangalore on 12.VIII.2012 (KDP), and eight adults were collected on 9–11.XI.2014 at Bangalore (H. M. Yeshwanth) on *Phoenix sylvestris*. Voucher specimens of *Javeta pallida* are deposited in the Kansas Natural History Museum, KS, USA, National Bureau of Agriculturally Important Insects, Bangalore, and the Travancore Insect Collection, Kerala Agricultural University, Vellayani, India. Vouchers of the parasitoids are deposited in the Zoological Survey of India, Western Ghats Regional Station, Kozhikode. A plant voucher of *Phoenix sylvestris* (accession no. 6863) is deposited in the Calicut University Herbarium, India.

## Results

**Life cycle of *Javeta pallida***. At Tirurangadi (field site 1), three palms of *Phoenix sylvestris* were observed heavily infested (Fig. 2) and with dried up older leaves. Eggs are laid singly mostly on the abaxial surface of leaves in longitudinal slits and are covered with a yellow secretion that turns reddish brown and forms an ootheca of about 1.8–2.3 mm length and 0.14–0.19 mm width (n=4) (Fig. 3). Freshly laid eggs, extracted from the slit of leaves, measured 1.35–1.38 mm in length and 0.25–0.28 mm in width (n=2), and were translucent yellow (Fig. 4). After the larva hatches, it bores into the mesophyll adjacent to the leaf cavity and initiates a leaf mine starting from the point of the egg insertion (Fig. 5). The leaf mines appear like elongate blotches of about 8.5–15.5 cm length and 0.5–1.1 cm width (n=18) (Fig. 6). Generally a single larva (Figs 7, 11, 12) occupies a mine, however, two or more larvae were also observed inside the mine when the adjacent larval mines of two or more individual larvae coalesce. A single leaflet of *Phoenix sylvestris* could support the development of up to four individuals. Pupation occurs inside the mine (Fig. 8). Fully mature larva, prior to pupation withdrew from the leading end of the leaf mine, where it is feeding, towards the middle of the mine and pupated. The pupa (Figs 8, 13, 14) exhibits little movement inside the hollow leaf mine, even when disturbed. However, when exposed by opening the leaf mine, it moves its abdomen vigorously and is also able to move forward and backward by applying the apex of its abdomen on the substrate, albeit to a limited extent. The adult emerged through an irregular hole of about 2.0–3.5 mm width (n=22) (Fig. 13). Adult emergence holes could be seen on either adaxial or abaxial surface of the leaf. Adults mostly feed on the abaxial surface of the leaflet making linear feeding trenches (Fig. 14). Generally they moved towards the tip of the leaves and feeding started from the apex to the base. Thus drying of leaves due to feeding starts from the apex of leaflets towards the petiole.

We observed the presence of larval mines and adult exit holes on 21 leaflets of the single *Phoenix dactylifera* at Tirurangadi (field site 2) in April, 2015, proving the occurrence of natural infestation of *Javeta pallida* on the date palm in Kerala, where it is of little commercial importance. A dead larva and pupal cases were recovered from the leaf mines, though no live insect was observed.

Adults confined on the first frond of *Phoenix dactylifera* during dry season at Tirurangadi (field site 3), laid 14 eggs. Twelve out of the 14 eggs hatched. Of the 12 larvae, nine pupated and finally emerged into adults. On the second frond 12 eggs were laid, however, only four of them hatched. Only one of the four larvae pupated and reached adulthood.

During the rainy season on the third frond of *Phoenix dactylifera*, ﻿22 eggs were laid and 18 of them hatched. Five of them reached pupal stage and all five emerged as adults. On the fourth frond, nine eggs were laid and all of them hatched. Of these nine larvae, five pupated and all emerged as adults.

On a wild date palm, *Phoenix sylvestris*,﻿ in the Botanical Garden of the University of Calicut (field site 4), we observed 21 eggs on a first frond during rainy season (second week of June, 2014 onwards). Nineteen of the 21 eggs hatched; 12 larvae pupated, and 12 adults emerged. On a second frond, we observed 29 eggs of which 24 hatched, 15 larvae pupated and 14 adults emerged; one pupa was observed dead inside the leaf mine. On a third frond, we observed 21 eggs; 19 hatched, and eventually 12 larvae reached pupal stage and adulthood.

Mature larvae and pupae often exited when the leaf mines were ruptured and such larvae pupated normally inside the glass beaker or nylon mesh in which they were confined and adults emerged.

A total of 58 adults were reared on *Phoenix dactylifera* and *Phoenix sylvestris*. However, the duration of all life stages from egg to adult could be tracked only in the case of 42 individuals, as at times the mines merged. Data on the developmental periods of *Javeta pallida* (based on the above 42 individuals), on *Phoenix dactylifera* during dry and rainy season as well as on both *Phoenix dactylifera* and *Phoenix sylvestris* during rainy season are presented in Table 1. Egg period on *Phoenix dactylifera* during dry season was significantly shorter than the same during the rainy season. Larval period also showed a similar trend, being highly significantly longer during rainy season than during the dry period. The pupal period was longer during rainy season, than during the dry season. However, the duration of pupal stage during dry and rainy seasons did not differ significantly on statistical comparison. The total developmental period was significantly longer on *Phoenix dactylifera* during rainy season (mean 71.63 days) compared to dry season (mean 58.7 days).

**Table 1.** Developmental periods of *Javeta pallida* on *Phoenix dactylifera* and *Phoenix sylvestris* during dry and rainy seasons.

Host	Season	No. of individuals tracked up to adulthood	Egg period (days)		Larval period (days)		Pupal period (days)		Total developmental period (days)	
			Mean	Standard deviation	Mean	Standard deviation	Mean	Standard deviation	Mean	Standard deviation
*Phoenix dactylifera*	Dry	10	12.2*	0.63	26.3*	3.59	20.2	1.32	58.7*	4.08
	Rainy	8	13*	0.93	37.5*	4.47	21.13	0.99	71.63*	5.15
*Phoenix sylvestris*	Rainy	24	13.04	1.0	34.33	5.85	20.45	1.32	67.83	6.53
t test	Dry vs Rainy seasons on *Phoenix dactylifera*	t value	2.18		5.90		1.645		5.946	
	*Phoenix dactylifera* vs *Phoenix sylvestris* in rainy season	t value	0.103		1.31		1.306		1.490	

* significantly different (5%)

During rainy season, egg, larval, pupal and total developmental periods of *Javeta pallida* on both *Phoenix sylvestris* and *Phoenix dactylifera* were statistically on par with each other.

In short, *Javeta pallida* completed development on *Phoenix* palms in 52–88 days (mean 66.38 days) with egg period 11–15 days (mean 12.8 days), larval period 21–54 days (mean 33.02 days) and pupal period 17–23 days (mean 20.52 days).

No beetles or signs of infestation were observed in commercial plantations of date palm in Dharmapuri, Tamil Nadu, during January or April, 2015.

**Figures 15–18.**
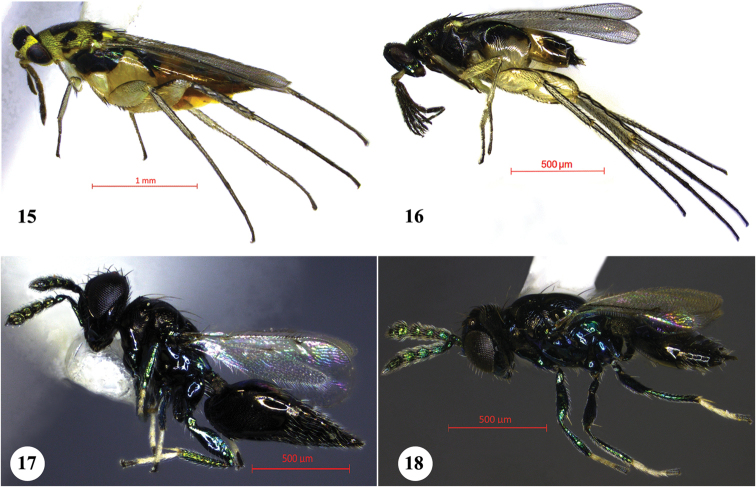
Parasitoids of *Javeta pallida*. **15** *Elasmus longiventris*, female **16** *Elasmus longiventris*, male **17** *Pediobius imbreus*, female **18** *Pediobius imbreus*, male.

**Natural enemies of *Javeta pallida***. Two species of chalcidoid parasitoids emerged from the larvae and/or pupae of *Javeta pallida* collected at Tirurangadi. Six females and six males of *Elasmus longiventris* Verma and Hayat (Figs 15, 16) and five females and 12 males of *Pediobius imbreus* Walker (Figs 17, 18) (both Eulophidae) emerged from larvae and/ or pupae in the laboratory.

## Discussion

The trophic selection of *Javeta pallida*, ﻿within Arecaceae, corresponds to that in other known members of the genus as well as most Coelaenomenoderini, as host plants of three *Javeta* species are previously known (Uhmann 1943, 1955; Kalshoven 1957, 1981).

The fundamental features of the life cycle of *Javeta pallida* follow the pattern in Coelaenomenoderini: single egg deposition, mining larvae with up to four instars, endogenous solitary pupation, and heavy infestation on the appropriate hosts. The female’s repertoire of making linear slits in the leaf, laying eggs singly within the slits, and then covering the egg firmly with brown colored material appears to be unique to *Javeta* within the tribe. In the most intensively studied Coelaenomenodera (Coelaenomenodera) elaeidis, ﻿females lay eggs in clusters at the ends of adult feeding scars and cover them with regurgitated leaf fibre (Cachan 1957; Howard et al. 2001; Mariau 2004). In Coelaenomenodera (Coelaenomenodera) lameensis eggs are laid in clusters inside cavities dugout on the leaf lamina and covered with faeces (Berti and Mariau 1999). We did not find groups of eggs, as has been noted for other Coelaenomenoderini — Coelaenomenodera (Coelaenomenodera) elaeidis (Cachan 1957; Morin and Mariau 1970, 1971, 1974; Mariau and Morin 1972, 1974) and Coelaenomenodera (Coelaenomenodera) lameensis (Berti and Mariau 1999; Mariau and Lecoustre 2000, 2004). In *Javeta pallida*, the slits in which eggs are laid, are independent of the adult feeding scars. There was no apparent additional covering, over ootheca, like frass as in other coelaenomenoderines. Laying single eggs probably is a better mechanism of defense against egg parasitoids than laying clusters of eggs in adult feeding scars. However, Kalshoven (1951) reported that 70% of the eggs of *Javeta arecae* were parasitized during an outbreak in April, 1937 in Sumatra, but he did not report the mode of oviposition in this species. The size of leaflet in *Phoenix sylvestris* (15–46 cm long, 2–2.5 cm wide) is less than that in oil palm (60–120 cm long, 3.5–5 cm wide). Smaller leaflet size in *Phoenix sylvestris* could be yet another driving factor behind *Javeta pallida* choosing solitary egg laying over egg clusters as this would ensure optimum availability of food resources for the larvae. In *Coelaenomenodera* spp., as a result of laying eggs in clusters, several larvae can occur within a single leaf mine (Cachan 1957). In *Javeta pallida*, a single larva per leaf mine is the norm, unless adjacent leaf mines coalesce. All of the Coelaenomenoderini life cycles documented to date indicate four larval instars. This is interesting as most Cassidinae have five instars and a few particular species have up to nine instars (Chaboo 2007).Coelaenomenoderine instar 1 appears to lack egg bursters (Cox 1988, 1994).

Drying of leaves due to adult feeding starts from the apex of leaflets towards the petiole. This appears to conserve the leaf as feeding near the base of the leaf lamina would result in drying up of the entire leaflet that could otherwise have been consumed.

At 74–97 days from egg to adult, the development of *Coelaenomenodera* spp. is relatively long among Cassidinae (Cotterell 1925; Morin and Mariau 1970; Appiah et al. 2007). These beetles appear to have high fecundity with females laying >70 eggs per week (Morin and Mariau 1971, 1974; Mariau and Bescombes 1972). Incubation is about 15–28 days, four larval stages last about 40–50 days, and pupation lasts up to 10–22 days (Cotterell 1925; Morin and Mariau 1970; Appiah et al. 2007). *Javeta pallida* that completes development in 52–88 days, too have a similar duration of life cycle. The data on duration of development of *Javeta pallida* during dry and rainy seasons on *Phoenix dactylifera* as well as during the rainy season on *Phoenix dactylifera* and *Phoenix sylvestris* present interesting patterns. The total developmental period and egg and larval periods were significantly longer during the rainy season than during the dry season, which indicates that dry climate is probably better for the growth and development of *Javeta pallida*. Similarly the near identical pattern of development of all life stages on both *Phoenix dactylifera* and *Phoenix sylvestris* indicates equal suitability of both host plants for beetle development. This suggests that outbreaks of *Javeta pallida* on the date palm is possible, as has happened on the wild date palm in Bangalore (Yeswanth H. M., personal communication) and Tirurangadi. Thus our rearing experiments have established the potential of *Javeta pallida* as a serious pest on the cultivated date palm.

Hymenoptera parasitoids belonging to the families Eulophidae and Trichogrammatidae act as the most important natural enemies of Coelaenomenoderini (Waterston 1925; Kerrich 1970, 1974; Boucek 1976; Viggiani 1980; Cox 1994; Mariau and Lecoustre 2004; Aneni 2014a, b). Morin and Mariau (1971) studied parasites and predators of the egg while Mariau et al. (1978) uncovered the parasites in each of the four larval instars. Discovery of two eulophid parasitoids on *Javeta pallida* reveal the same pattern of host-parasite relationship.

Although some papers have been titled “morphology” they give only minimal information about morphological structures. Therefore detailed comparative study of all life stages, including scanning electron microscopy, is needed both to uncover many more taxonomic and phylogenetic characters to strengthen understanding of systematics and evolution and to better manage a notorious economically-important pest.

One of the most remarkable aspects of Coelaenomenoderini life cycles is the alternation of mixed populations of different stages with synchronized populations in outbreak periods. This has been described for Coelaenomenodera (Coelaenomenodera) elaeidis (Mariau and Morin 1972; Bernon and Graves 1979) and Coelaenomenodera (Coelaenomenodera) lameensis (Mariau and Lecoustre 2004). There are many such sporadic pests, such as the rice caseworm, *Nymphula depunctalis* (Guenee) (Lepidoptera: Pyralidae), rice swarming caterpillar, *Spodoptera mauritia* (Boisduval) (Lepidoptera: Noctuidae), and locusts having periodic swarms and outbreaks. It is unclear at this time what factors trigger the changes in life cycles (from asynchronous to synchronous) and what might be any behavioral, morphological and physiological changes. Despite the serious pest status of these species, very little has been written about the natural history of the adults.

Several factors contribute to their success. The females have very high fecundity (for Cassidinae) and there can be up to four generations per year (Timti 1991). Distinct cycles with periodic outbreaks have been documented for Coelaenomenodera (Coelaenomenodera) elaeidis (Morin and Mariau 1970; Bernon and Graves 1979; Mariau et al. 1999a; Mariau and Lecoustre 2004), and Coelaenomenodera (Coelaenomenodera) lameensis (Berti and Mariau 1999). The scraping and mining behavior of feeding produces severely damaged leaves and defoliated trees; this results in costly lower yields of fruit and oil. Chemical (Jover 1950; Mariau et al. 1973, 1979; Philippe and Diarrassouba 1980; Mariau and Philippe 1983; Philippe 1990), host plant resistance (Mariau et al. 1999b) and parasitoid (Mariau and Morin 1972; Mariau et al. 1978; Lecoustre et al. 1980) control measures must be well-timed for this phasic pattern in population explosions.

The severity of infestation observed on *Phoenix sylvestris* suggests that *Javeta pallida* poses a potential pest of any species of *Phoenix*, including *Phoenix dactylifera*. Our study foresees a native leaf beetle becoming a serious pest on an exotic crop of immense economic potential in India.

Chemical control with sprays and injection of trees have been used to control *Coelaenomenodera* pests (Jover 1950; Philippe 1990; Mariau et al. 1973, 1979; Mariau and Genty 1992). However, Timti (1991) indicated that years of chemical sprays had little effect in controlling infestations of Coelaenomenodera (Coelaenomenodera) elaeidis in West and Central Africa. Chemical measures may also have limited use against the larvae concealed inside mines. Alternative control measures must be developed for long term control. Limited data suggests that palm hybrids with different leaf mechanical properties can impede larval development (e.g. Mariau et al. 1999a).

World-wide interest in more sustainable and healthier harvesting and processing of food is stimulating shifts to organic farming, including in date-palm and wild date palms (Mahmoudi et al. 2008). This change of attitude and its economic implications suggest that biological control measures that exploit the predators and parasitoid complex of each life stage of Coelaenomenoderini may be the most economical, most effective, and most sustainable long-term control. Eulophidae and Trichogrammatidae can parasitize all life stages, including eggs and larvae that are encased within the leaf. Timti’s (1991) study with Coelaenomenodera (Coelaenomenodera) elaeidis populations in Cameroon revealed that ants can also act as biocontrols. These studies carried out in Africa more than 30 years ago may provide a model to pursue knowledge about the Indian parasitoid complex if *Javeta* becomes a major pest of expanding indigenous and introduced palm crops.

**Conclusion.** Comparative study of morphology and biology across Coelaenomenoderini will certainly yield many novel phylogenetic characters. Our study here suggests that the oviposition (number and coverage of eggs), number of instars and morphology, pupation site, and eruptive population behaviors might be considered as character complexes. Palms are one of the most important crops in the world and their insect fauna needs further study. Within Cassidinae, we also need to understand the evolutionary relationship of certain tribes with palms. Furthermore, study of the insect milieu—the predator and parasitoid complex—can help us understand their impacts on the beetles’ evolution and provide models for sustainable biocontrols of palm resources. We plan to continue documenting the biology, pest status, and insect enemy complex of *Javeta pallida* in the field. Our next step is also a detailed morphological study of the juvenile and adult stages.
